# Supplementary material for: Genome-Wide Identification and Salt Stress Response Analysis of the bZIP Transcription Factor Family in Sugar Beet
Source: Int J Mol Sci. 2022 Sep 30;23(19):11573. doi: 10.3390/ijms231911573 (PMC9569505; doi:10.3390/ijms231911573)
Supplement: Supplementary file 1 [file ijms-23-11573-s001.zip › ijms-1866893-supplementary.pdf]

Supplement Table S1. Basic information of the *BvbZIP* genes family.

Gene name	Gene ID	Chromosome	Genomic location	Protein ID	Length(aa)	*p*I	MW(kDa)
BvbZIP1	LOC104905689	Chr1	501404-512443	BvbZIP1a	360	6.78	40.87
				BvbZIP1b	360	6.78	40.87
BvbZIP2	LOC104886686	Chr1	3977916-3982942	BvbZIP2a	420	6.16	44.61
				BvbZIP2b	420	6.16	44.61
				BvbZIP2c	418	6.16	44.38
BvbZIP3	LOC104887833	Chr1	5540972-5546329	BvbZIP3	532	6.27	58.42
BvbZIP4	LOC104889396	Chr1	7653114-7654814	BvbZIP4	171	5.2	19.58
BvbZIP5	LOC104895660	Chr1	18474752-18486050	BvbZIP5	361	8.03	39.30
BvbZIP6	LOC104895875	Chr1	19888225-19896453	BvbZIP6	332	6.64	36.63
BvbZIP7	LOC104899269	Chr1	29718507-29722385	BvbZIP7	305	4.78	34.71
BvbZIP8	LOC104905249	Chr2	2777401-2779008	BvbZIP8	269	6.06	29.66
BvbZIP9	LOC104907325	Chr2	5556010-5557185	BvbZIP9	184	5.25	21.45
BvbZIP10	LOC104885558	Chr2	10954314-10961255	BvbZIP10	841	5.73	92.44
BvbZIP11	LOC104886702	Chr2	15713633-15722655	BvbZIP11a	351	5.99	38.47
				BvbZIP11b	350	5.99	38.34
BvbZIP12	LOC104887563	Chr2	36849298-36858494	BvbZIP12a	455	6.16	50.10
				BvbZIP12b	397	7.08	44.18
BvbZIP13	LOC104887633	Chr2	37909453-37913193	BvbZIP13	406	5.36	45.71
BvbZIP14	LOC104887661	Chr2	38413121-38417226	BvbZIP14	168	9.9	18.15
BvbZIP15	LOC104888155	Chr3	3093992-3095517	BvbZIP15	173	6.13	19.53
BvbZIP16	LOC104888309	Chr3	4903671-4911186	BvbZIP16a	348	6.72	37.28
				BvbZIP16b	348	6.72	37.28
				BvbZIP16c	348	6.72	37.28
				BvbZIP16d	345	6.72	36.92
				BvbZIP16e	348	6.72	37.28
BvbZIP17	LOC104888416	Chr3	6266841-6267350	BvbZIP17	169	8.81	18.89
BvbZIP18	LOC104888846	Chr3	12517919-12529117	BvbZIP18	388	6.02	42.86
BvbZIP19	LOC104889231	Chr3	19135726-19150319	BvbZIP19	577	7.17	63.77
BvbZIP20	LOC104889491	Chr3	23089301-23097287	BvbZIP20a	449	7.12	49.51
				BvbZIP20b	426	6.05	47.46
BvbZIP21	LOC104889531	Chr3	23723459-23726886	BvbZIP21	690	5.66	75.24
BvbZIP22	LOC104889671	Chr3	25013663-25018273	BvbZIP22a	333	9.27	36.54
				BvbZIP22b	333	9.27	36.54
BvbZIP23	LOC104906977	Chr4	459791-463907	BvbZIP23	164	5.47	19.01
BvbZIP24	LOC104907452	Chr4	1102915-1103742	BvbZIP24	275	5.03	30.80
BvbZIP25	LOC104890911	Chr4	12442060-12447944	BvbZIP25	435	6.47	47.16
BvbZIP26	LOC104891424	Chr4	24868943-24873916	BvbZIP26a	353	5.73	38.59
				BvbZIP26b	352	5.73	38.51
BvbZIP27	LOC104893043	Chr5	16232097-16244958	BvbZIP27a	397	5.85	42.14
				BvbZIP27b	395	5.97	41.89
				BvbZIP27c	371	5.85	39.44
BvbZIP28	LOC104894764	Chr5	51617619-51618983	BvbZIP28	141	5.87	16.21
BvbZIP29	LOC104894976	Chr6	1714961-1716254	BvbZIP29	289	8.49	32.88
BvbZIP30	LOC104895073	Chr6	2809761-2811097	BvbZIP30	180	5.36	20.24
BvbZIP31	LOC104895559	Chr6	8140566-8161505	BvbZIP31a	240	9.35	26.69
				BvbZIP31b	239	8.36	26.66
BvbZIP32	LOC104895725	Chr6	10485448-10490260	BvbZIP32a	363	6.12	41.23
				BvbZIP32b	327	5.3	36.85
BvbZIP33	LOC104896277	Chr6	20681507-20682236	BvbZIP33	204	6.17	23.95

BvbZIP34	LOC104896274	Chr6	20809273-20810261	BvbZIP34	200	5.56	23.18
BvbZIP35	LOC104898371	Chr7	3838768-3848663	BvbZIP35	451	8.3	49.19
BvbZIP36	LOC104898393	Chr7	4346206-4351489	BvbZIP36	465	6.87	51.64
BvbZIP37	LOC104899695	Chr7	35447527-35451803	BvbZIP37	489	9.49	51.59
BvbZIP38	LOC104900287	Chr7	43158294-43161957	BvbZIP38	386	5.69	42.52
BvbZIP39	LOC104900318	Chr7	43480497-43490642	BvbZIP39a	514	7.84	57.29
				BvbZIP39b	514	7.84	57.29
				BvbZIP39c	510	8.3	56.97
				BvbZIP39d	500	7.1	55.79
				BvbZIP39e	485	8.66	53.93
BvbZIP40	LOC104900999	Chr8	8997688-8998614	BvbZIP40	208	5.76	23.84
BvbZIP41	LOC104883107	Chr9	13148-16084	BvbZIP41	572	6.94	62.25
BvbZIP42	LOC104903082	Chr9	11871350-11881962	BvbZIP42a	281	8.42	31.34
				BvbZIP42b	276	8.23	30.70
				BvbZIP42c	270	6.48	30.11
				BvbZIP42d	264	6.92	29.46
				BvbZIP42e	248	7.63	27.45
				BvbZIP42f	245	8.55	27.17
				BvbZIP42g	281	8.42	31.34
				BvbZIP42h	257	8.24	28.66
BvbZIP43	LOC104903424	Chr9	21452927-21456740	BvbZIP43	349	5.67	39.18
BvbZIP44	LOC104903923	Chr9	33644446-33649387	BvbZIP44	290	5.89	31.71
BvbZIP45	LOC104904696	Chr9	41736456-41743229	BvbZIP45	380	8.65	42.46
BvbZIP46	LOC104884020	Unknown	5540-16672	BvbZIP46a	205	5.71	23.55
				BvbZIP46b	194	6.6	21.85
BvbZIP47	LOC104884708	Unknown	83055-94429	BvbZIP47	353	5.23	37.06
BvbZIP48	LOC104884843	Unknown	52251-61547	BvbZIP48a	434	6.06	48.48
				BvbZIP48b	429	6.02	47.83

Supplement Table S2. SRA transcriptome data item information.

Sample ID	Accession	Treatment	Time	Tissue
SRR12730708	PRJNA666117	Salt stress（300 mM NaCl）	control（0 h）	Leaf
SRR12730707	PRJNA666117	Salt stress（300 mM NaCl）	control（0 h）	Leaf
SRR12730696	PRJNA666117	Salt stress（300 mM NaCl）	control（0 h）	Leaf
SRR12730685	PRJNA666117	Salt stress（300 mM NaCl）	12 h	Leaf
SRR12730684	PRJNA666117	Salt stress（300 mM NaCl）	12 h	Leaf
SRR12730683	PRJNA666117	Salt stress（300 mM NaCl）	12 h	Leaf
SRR12730682	PRJNA666117	Salt stress（300 mM NaCl）	24 h	Leaf
SRR12730681	PRJNA666117	Salt stress（300 mM NaCl）	24 h	Leaf
SRR12730680	PRJNA666117	Salt stress（300 mM NaCl）	24 h	Leaf
SRR12730679	PRJNA666117	Salt stress（300 mM NaCl）	48 h	Leaf
SRR12730706	PRJNA666117	Salt stress（300 mM NaCl）	48 h	Leaf
SRR12730705	PRJNA666117	Salt stress（300 mM NaCl）	48 h	Leaf
SRR12730704	PRJNA666117	Salt stress（300 mM NaCl）	72 h	Leaf
SRR12730703	PRJNA666117	Salt stress（300 mM NaCl）	72 h	Leaf
SRR12730702	PRJNA666117	Salt stress（300 mM NaCl）	72 h	Leaf
SRR12730701	PRJNA666117	Salt stress（300 mM NaCl）	control（0 h）	Root
SRR12730700	PRJNA666117	Salt stress（300 mM NaCl）	control（0 h）	Root
SRR12730699	PRJNA666117	Salt stress（300 mM NaCl）	control（0 h）	Root
SRR12730698	PRJNA666117	Salt stress（300 mM NaCl）	12 h	Root
SRR12730697	PRJNA666117	Salt stress（300 mM NaCl）	12 h	Root

SRR12730695	PRJNA666117	Salt stress（300 mM NaCl）	12 h	Root
SRR12730694	PRJNA666117	Salt stress（300 mM NaCl）	24 h	Root
SRR12730693	PRJNA666117	Salt stress（300 mM NaCl）	24 h	Root
SRR12730692	PRJNA666117	Salt stress（300 mM NaCl）	24 h	Root
SRR12730691	PRJNA666117	Salt stress（300 mM NaCl）	48 h	Root
SRR12730690	PRJNA666117	Salt stress（300 mM NaCl）	48 h	Root
SRR12730689	PRJNA666117	Salt stress（300 mM NaCl）	48 h	Root
SRR12730688	PRJNA666117	Salt stress（300 mM NaCl）	72 h	Root
SRR12730687	PRJNA666117	Salt stress（300 mM NaCl）	72 h	Root
SRR12730686	PRJNA666117	Salt stress（300 mM NaCl）	72 h	Root

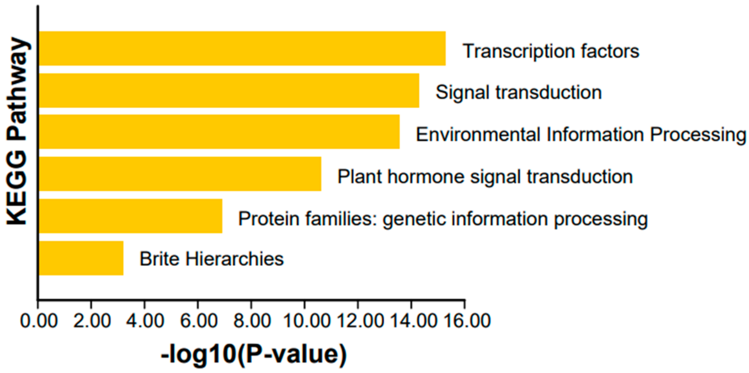

Supplement Figure S1. KEGG enrichment analysis of *BvbZIP* genes. The *BvbZIP* genes was enriched into six KEGG pathways, including environmental information processing and plant hormone signal transduction processes.

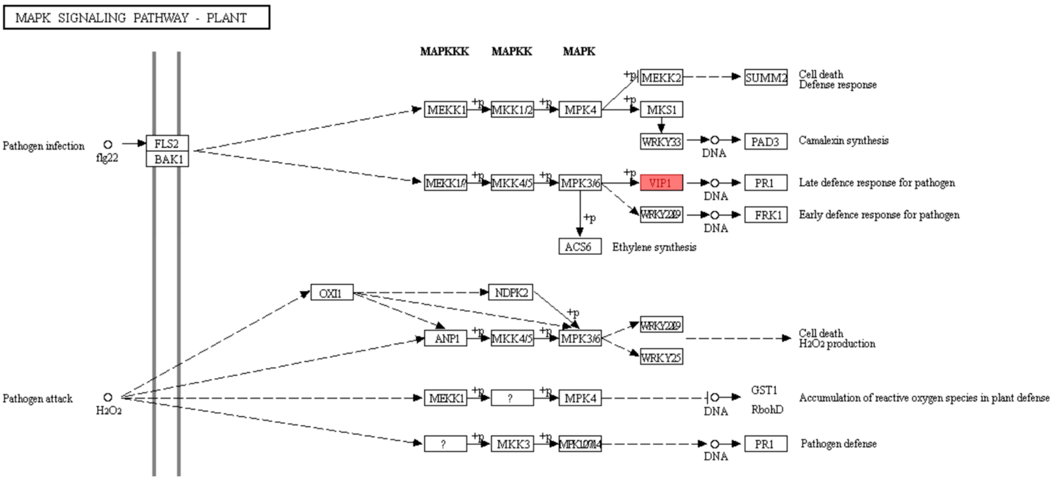

Supplement Figure S2. KEGG pathway of *BvbZIP3*(VIP1) gene. *BvbZIP3*(VIP1) is involved in the cascade reaction pathway of pathogen infection and plays a regulatory role in late infection defense.

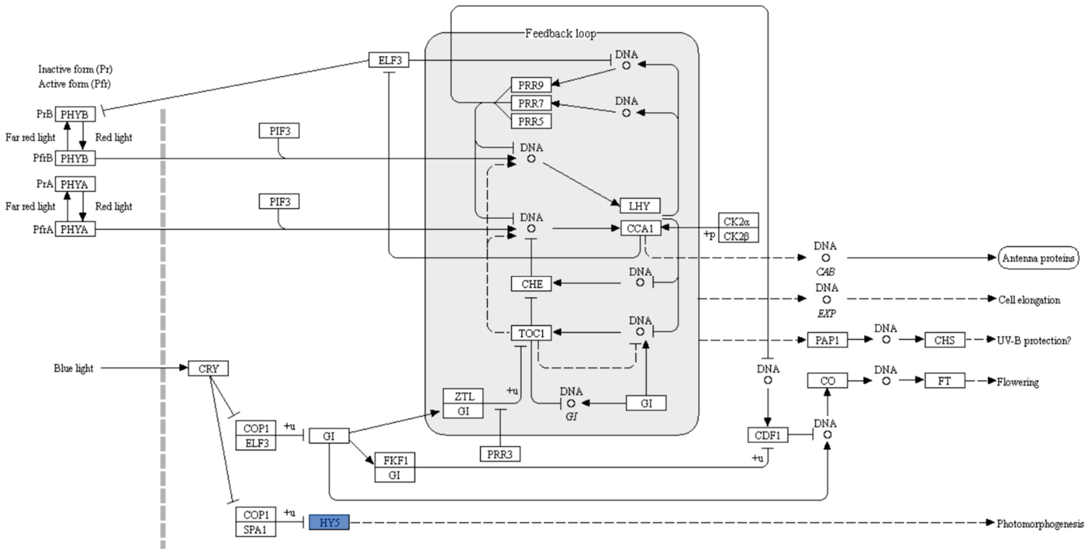

Supplement Figure S3. KEGG pathway of *BvbZIP14*(HY5) gene. *BvbZIP14*(HY5) is a downstream gene of COP1 and SPA1, which is involved in the blue light response pathway.

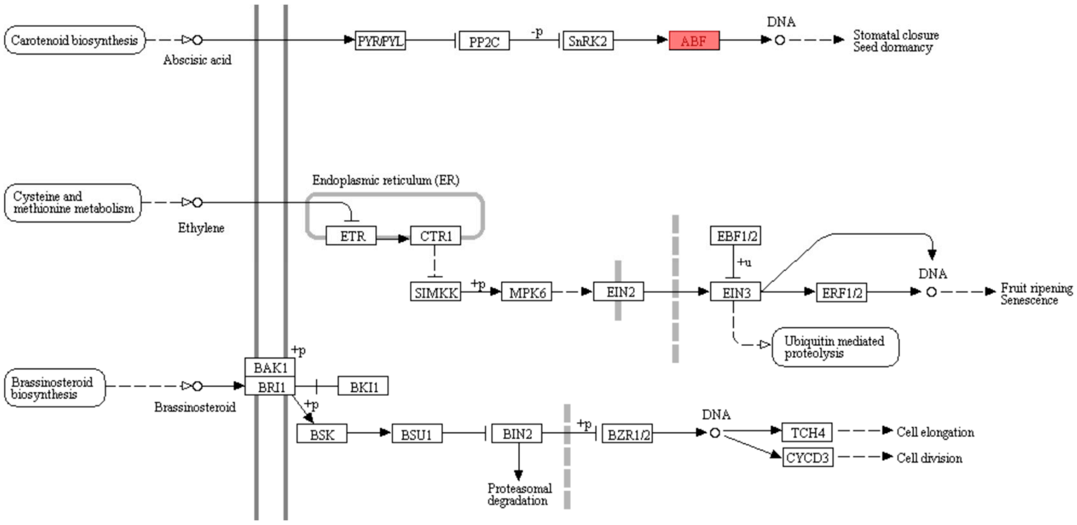

Supplement Figure S4. KEGG pathway diagram of *BvbZIP37*(ABF) gene. *BvbZIP37*(ABF) regulates stomatal closure and seed dormancy in response to ABA signals.
